# Reconstructing Interventional Cardiology Fellowships to Include Cardiac Computed Tomography Training

**DOI:** 10.1016/j.jscai.2023.101057

**Published:** 2023-06-25

**Authors:** Scott E. Janus, Tarek Chami, Shilpkumar Arora, Mario Goessl, Paul Sorajja, Steven J. Filby, Neal S. Kleiman, Mehdi H. Shishehbor, John T. Saxon, Emmanouil S. Brilakis, Sadeer G. Al-Kindi

**Affiliations:** a St Luke’s Mid America Heart Institute, Kansas City, Missouri; b University of Missouri-Kansas City, Kansas City, Missouri; c Minneapolis Heart Institute, Abbott Northwestern Hospital, Minneapolis, Minnesota; d Department of Cardiology, Houston Methodist DeBakey Heart & Vascular Center, Houston, Texas; e Harrington Heart and Vascular Institute, University Hospitals Cleveland Medical Center, Cleveland, Ohio

**Keywords:** cardiac computed tomography, Core Cardiology Training Symposium, fellowship training

Cardiovascular imaging is essential for the evaluation and treatment of cardiovascular disease in the cardiac catheterization laboratory. The evolution and innovations in cardiac computed tomography (CCT) including applications in coronary, congenital, structural, and aortic/vascular pathologies have made this modality integral to the interventional cardiologist’s repertoire.^1^ Yet, many interventional cardiologists, including recent graduates of fellowship programs, lack basic experience in interpreting and understanding CCT.^2^ With the recent release of the 2023 American College of Cardiology (ACC)/American Heart Association (AHA)/Society for Cardiovascular Angiography & Interventions (SCAI) Advanced Training Statement,^3^ Society of Cardiovascular Computed Tomography preprocedural guidelines,^4^ and clinical practice guidelines for chest pain evaluation, recommending CCT with multiple Class 1 and 2 indications,^5^ we call for the integration of a dedicated CCT curriculum within interventional cardiology training.

The utilization of CCT not only improves diagnostic accuracy in identifying acute pathologies (pulmonary embolism, dissections, coronary artery occlusions)^6^ but has become essential for preprocedural management. In the United States, the 2023 Advanced Training Statement missed an opportunity to properly address CCT imaging for interventional fellowships, which is increasingly being utilized by interventional cardiologists for procedural planning and follow-up.

To assess the current trends and needs for interventional cardiology fellows, we surveyed individuals at 2 interventional cardiology courses designed for current fellows in 2022: the Cardiovascular Innovations course in Denver, Colorado and SCAI Interventional Fellows Course in Miami, Florida. An anonymous survey was distributed that contained 11 questions regarding fellows’ viewpoints on CCT curriculum and needs in an interventional cardiology fellowship. Survey questions assessed in scale (1) how often CCT is used in preprocedural planning (coronary compared with peripheral/structural), (2) how comfortable fellows are interpreting CCT (on a scale of 0 to 5, with 0 indicating not at all comfortable, and 5 indicating very comfortable), and (3) the fellows’ current Core Cardiology Training Symposium (COCATS) level of CCT training.

Overall, 77 surveys were completed. Approximately half (n = 40, 52%) were current US-based interventional cardiology fellows. The remainder consisted of international fellows (n = 37, 48%). The majority of respondents were male (n = 63, 82%) and in academic centers (n = 55, 71%). Overall, 42 (55%) designated themselves as COCATS Level 0 for CCT knowledge, and 19 (25%) currently have CCT teaching within their fellowship program. The majority (n = 40, 52%) have interpreted 0 to 50 CCT scans during their training, with only 8 (10%) having interpreted over 250 CCT scans. Thirty-two (42%) responders felt uncomfortable or only mildly comfortable interpreting for structural procedures, whereas 27 (35%) felt uncomfortable or mildly comfortable interpreting computed tomography (CT) for coronary procedures. The majority of respondents (n = 66, 86%) wished they had dedicated time/teaching to CT within their interventional fellowship program.

Despite the relatively low percentage of fellows who have dedicated training in CT during interventional cardiology fellowship, it is clear CT training confers a number of advantages in clinical practice. CT angiography is the optimal imaging modality to evaluate the appropriate access sites and reduce the risk of common vascular complications. Having the ability to quickly interpret coronary trifurcations, native ostial disease, bypass graft course, and anomalous coronary origins is paramount to achieving optimal results and care for each patient.

**Figure 1:**
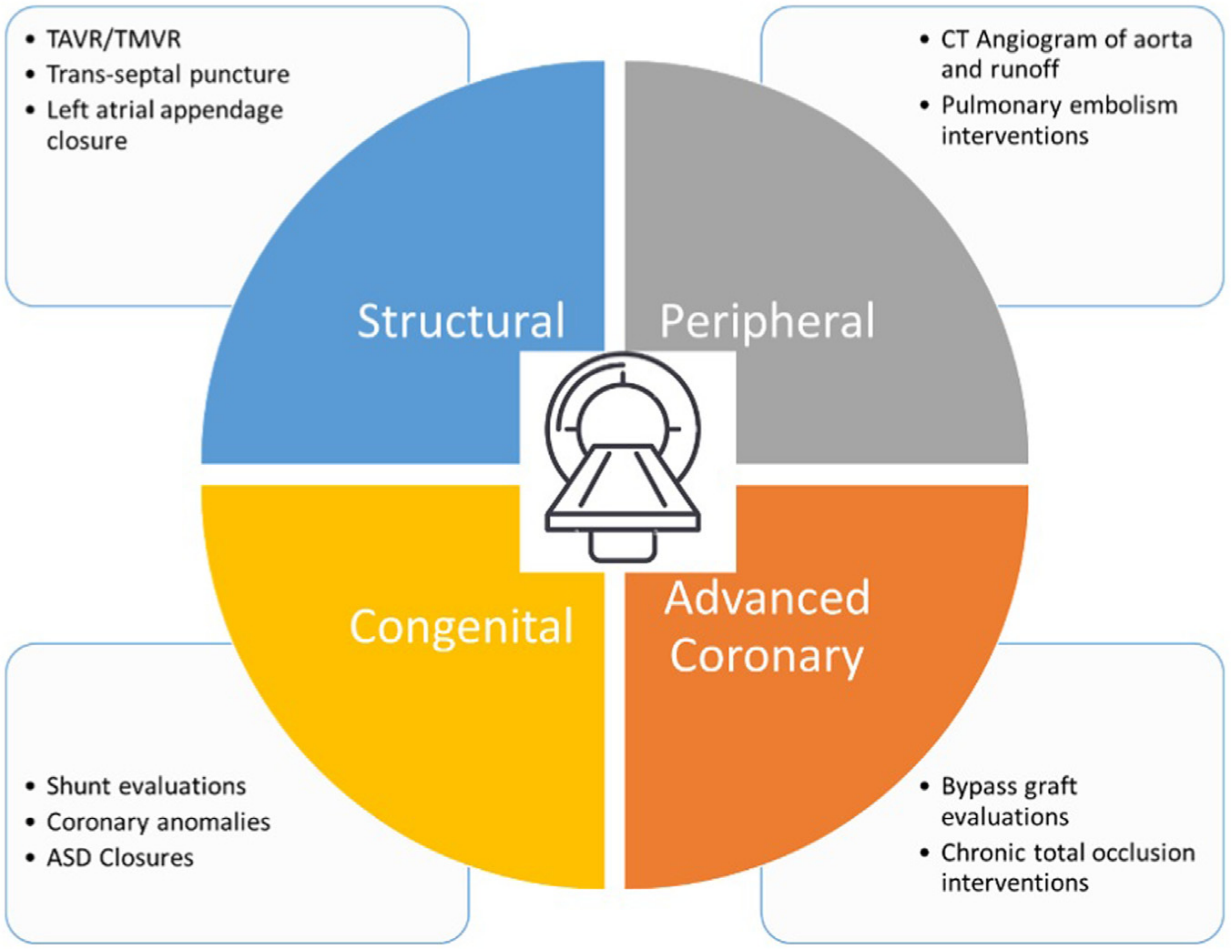
**Current CT usage among interventional specialties.** ASD, atrial septal defect; CT, computed tomography; TAVR, transcatheter aortic valve replacement, TMVR, transcatheter mitral valve replacement.

Furthermore, individuals pursuing the subspecialty fields of interventional cardiology (structural, advanced coronary, peripheral) not only benefit from CT training but require it for optimal practice (Figure 1). In structural interventions, accurate sizing of the aortic annulus was the first clinically relevant application of CT in transcatheter aortic valve replacement, dramatically impacting the incidence of paravalvular leak after the procedure.^7^ Additionally, for individuals training in complex and high-risk coronary interventional procedures assessment of collateral circulation, anatomical course of the occlusion segment, and characterization of distal and proximal cap anatomy, CT augments their training. Peripheral vascular fellows benefit from extensive knowledge of CT angiography of the carotid arteries and aorta for determination of the appropriateness of percutaneous interventions and to determine optimal access, along with evaluation of side branches for percutaneous aortic interventions (eg, endovascular aortic repair, and thoracic endovascular aneurysm repair).

Several steps are needed to address the lack of CCT training for future interventional cardiologists. First, formalized standards along with designated and mentored teaching of CCT imaging need to be investigated and included in the next iterations of COCATS. Although expert consensus statements have been published for structural heart disease interventions,^8^ we call for these to be adapted for first year interventional cardiology and recognized in the consensus training statement. We propose formal integration of career-specific CT training in interventional fellowship as well as the incorporation of CCT imaging requirements into the COCATS training statement for interventional cardiology and the advanced training statements. We additionally propose dedicated time during fellowship for image interpretation with experienced cardiac imaging physicians to review CCT scans. A more streamlined approach would be to include Level 1 or 2 CT training in general cardiology fellowship for fellows who express an early interest in interventional fellowship, which may afford the same CT experiences for the trainee without compromising procedural training. Incorporation of CT-based questions in the interventional cardiology board certification examinations will additionally incentivize interventional cardiology fellows to gain experience in CT. By implementing these measures, we hope all graduating interventional fellows can achieve at least COCATS Level 1 and ideally Level 2 training in CCT to render more specialized care for patients.

Therefore, we call on leaders in the interventional community, SCAI/ACC and/or the Accreditation Council for Graduate Medical Education to encourage the incorporation of CCT training into formal guidelines with a minimum number of interpreted scans (similar to percutaneous interventions) to bridge the competency gap and equip future interventional cardiologists with the necessary tools to be successful. By addressing the current limitations, we hope to improve education across interventional fellowships and further fuse the innovations in imaging and intervention.
